# Utility of medical record diagnostic codes to ascertain attention-deficit/hyperactivity disorder and learning disabilities in populations of children

**DOI:** 10.1186/s12887-020-02411-3

**Published:** 2020-11-07

**Authors:** Yu Shi, Phillip J. Schulte, Andrew C. Hanson, Michael J. Zaccariello, Danqing Hu, Sheri Crow, Randall P. Flick, David O. Warner

**Affiliations:** 1 Department of Anesthesiology and Perioperative Medicine, Mayo Clinic, 200 1st St SW, Rochester, MN 55905 USA; 2 Department of Health Sciences Research, Mayo Clinic, Rochester, USA; 3 Department of Psychiatry and Psychology, Mayo Clinic, Rochester, USA; 4 Department of Pediatric and Adolescent Medicine, Mayo Clinic, Rochester, USA

**Keywords:** Attention-deficit/hyperactivity disorder, Learning disability, Diagnostic code, Machine learning, Validation

## Abstract

**Background:**

To develop and evaluate machine learning algorithms to ascertain attention-deficit/hyperactivity (ADHD) and learning disability (LD) using diagnostic codes in the medical record.

**Method:**

Diagnoses of ADHD and LD were confirmed in cohorts of children in Olmsted County of Minnesota based on validated research criteria. Models to predict ADHD and LD were developed using ICD-9 codes in a derivation cohort of 1057 children before evaluated in a validation cohort of 536 children.

**Results:**

The ENET-MIN model using selected ICD-9 codes at prior probability of 0.25 has a sensitivity of 0.76, PPV of 0.85, specificity of 0.98, and NPV of 0.97 in the validation cohort. However, it does not offer significant advantage over a model using a single ICD-9 code of 314.X, which shows sensitivity of 0.81, PPV of 0.83, specificity of 0.98, and NPV of 0.97. None of the models developed for LD performed well in the validation cohort.

**Conclusions:**

It is feasible to utilize diagnostic codes to ascertain cases of ADHD in a population of children. Machine learning approaches do not have advantage compared with simply using a single family of diagnostic codes for ADHD. The use of medical record diagnostic codes is not feasible to ascertain LD.

**Supplementary Information:**

The online version contains supplementary material available at 10.1186/s12887-020-02411-3.

## Background

Neurodevelopmental disorders such as attention-deficit/hyperactivity disorder (ADHD) and learning disabilities (LD) can have a long-term impact on learning as well as emotional, behavioral and social development [1, 2]. Knowledge of the incidence and prevalence of such neurodevelopmental disorders is crucial for formulating public health policies that can benefit these children, such as special education funding. The analysis of health administrative data can be a valuable tool in studying neurodevelopmental disorders. Such data enables population-level analysis, minimizes selection bias, improves generalizability, and allows accurate estimation of disease incidence and prevalence [3]. In addition, data are often readily accessible at relatively low cost, permitting feasible and timely analysis [4]. However, because health administrative data such as diagnostic codes are collected for billing and administrative purposes, not research, they require validation before being utilized in public health research [3]. For example, the accuracy of diagnostic codes in identifying patients with or without a specific condition depends on the quality of the data and the condition of interest [5]. It is also possible that for a given outcome, not all relevant information required for ascertainment may be consistently available in the medical record.

The aim of this study was to develop and evaluate machine learning algorithms to ascertain ADHD and LD using diagnostic codes in the medical record. In prior work, we constructed a population-based birth cohort to study the relationship between early anesthesia exposure and developmental outcomes in children, including ADHD and LD [6]. In this work, the diagnoses of ADHD and LD were evaluated by in-depth manual review of both clinical and school records using validated research criteria. This dataset provides the opportunity to examine the relationship between information available from medical record diagnostic codes and the “gold standard” of diagnoses by validated research criteria which also incorporate information from school records. We hypothesized that machine learning models could be developed using diagnostic codes in the medical charts to ascertain ADHD and LD diagnoses in our cohort. Unpublished work by members of our study team using a separate birth cohort has examined the development of ADHD and LD after care in the pediatric intensive care unit (ICU), again using review of both clinical and school records. This provided a separate cohort to validate our models derived from the first cohort. Previous publications evaluated the accuracy of ICD codes in defining ADHD by manual review of clinical charts [7, 8]. Our work was the first to utilize validated criteria to ascertain ADHD beyond the information from clinical record. To our knowledge, machine learning models have not yet been applied to ascertaining of ADHD or LD.

## Methods

### Study cohorts

The derivation cohort was a propensity-matched cohort examining the association between anesthetic exposure and subsequent neurodevelopmental outcomes, including LD and ADHD [6]. In summary, a birth cohort of all children born in Olmsted County, MN from January 1, 1996 to December 31, 2000 was identified. For each child, school enrollment status in the local public school district at age 5 and all episodes of anesthetic exposure before age 3 were identified. The derivation cohort was created by selecting children enrolled in the school district (and thus survived and were resident in Olmsted county until at least age 5) based on their propensity to receive general anesthesia, using multiple variables including information from birth certificates and medical diagnoses to calculate the propensity score. Children were followed up to December 31, 2014.

The validation cohort was generated as part of an unpublished study to study the association of pediatric ICU admission and neurodevelopmental outcomes. A population-based birth cohort included children born in Olmsted County, MN during a 5 year period (1/1/2003–12/31/2007) with an ICU admission prior to age 4. Each child with an ICU admission was matched (based on gender, birth date (± 30 days), maternal age (± 3 years) and education level) with a child who were not admitted to the ICU prior to age 4. These children were followed for up to 11 years after ICU admission (last follow up date 12/31/2013).

All diagnostic codes from birth were available for all cohort members through the Rochester Epidemiology Project, a population-based medical records linkage system [9]. For each outcome, a master list of all International Classification of Diseases (ICD)-9 codes received by each child during his/her lifetime was generated in chronologic order. A list for further analysis was then generated from the master list by deleting all duplicated ICD-9 codes, resulting in all distinct ICD-9 codes received by each child during their lifetimes.

### Diagnostic criteria for ADHD and LD

Cases of ADHD and LD were ascertained by study personnel by manually review of clinical and school records of all children in the cohorts.

#### Attention-deficit hyperactivity disorder (ADHD)

ADHD cases were defined based on criteria previously described [10]. The criteria rely on documentation within medical and school records of ADHD diagnoses and questionnaires completed by caregivers. Children were identified as ADHD cases if their records included either 1) a clinical diagnosis in the medical record with accompanying description of appropriate symptoms, or; 2) a positive ADHD questionnaire in the school record. ADHD questionnaire results were considered positive only when both parent and teacher questionnaires were positive. The exclusion criteria specified in the Diagnostic and Statistical Manual of Mental Disorders (Fourth Edition) (DSM-IV) were followed (i.e., ADHD symptoms were not better accounted for by a mood disorder, psychotic disorder, schizophrenia, severe intellectual disability, or pervasive developmental disorder). DSM-IV criteria for ADHD were not otherwise used [10].

#### Learning disabilities (LD)

LD cases were ascertained according to previously-described research criteria based on one of two formulas: an intelligence quotient (IQ)-achievement discrepancy formula and a low achievement formula [11]. Children were considered to have LD if they met research criteria for at least one of the three LD subtypes (reading, written language, and mathematics disabilities) determined by either of the formulas using contemporaneous IQ and achievement scores.

### Classification algorithms

For each outcome (ADHD or LD), we aimed to identify classifier algorithms with optimized predictive ability by comparing algorithm results with the confirmed cases. Machine learning models were developed and trained in the derivation cohort before applied to the validation cohort.

We considered four methods for classification: Least Absolute Shrinkage and Selection Operator (LASSO) logistic regression, Elastic Net (ENET) logistic regression, Classification trees (CART), and Stochastic Gradient Boosting (GBM). Inputs included ICD-9 diagnosis codes. To summarize, LASSO and ENET are regression analysis methods that perform both variable selection and regularization in order to enhance the prediction accuracy and interpretability of the statistical model produced. CART and GBM are tree-based methods, with the latter using boosting to combine many weak classifiers into a single strong classifier. Each method further implemented internal cross-validation selection of tuning parameters that affect model complexity of the classifier based on the misclassification error. For each method, we considered tuning parameters minimizing cross-validation mean misclassification error (MIN) and one maximizing regularization or pruning but still within 1 standard error (1SE) of the minimum misclassification error which may reduce overfitting.

Because the prevalence of cases in the cohort is relatively low, the number of cases is less than the number of non-cases. By minimizing misclassification error misclassification is roughly equally likely to occur in those predicted/classified as case and non-case status; as a result, we would expect a higher proportion of true cases will be misclassified as compared to that of non-cases, leading to low sensitivity and high specificity. One approach to overcome this sensitivity/specificity imbalance is assigning more weight to the cases during model training, thereby increasing the cost of misclassifying a case as compared to a non-case. Several case-weight options were considered in the derivation dataset, modeling prior probabilities for cases ranging from 10 to 75% (representing the proportion of LD or ADHD cases after different weights are assigned).

For each of these methods, additional factors were considered. The first was whether to include within the classifier all ICD-9 codes or a subset of codes that are plausibly related to the diagnosis in question. The former approach makes no presuppositions and uses all data, but may also increase the possibility of spurious associations or overfitting, especially as the number of events within the dataset is relatively modest and substantially less than the number of codes considered (3597). The latter approach may increase specificity, but may also miss important unanticipated associations. For the latter, experts in pediatric neurodevelopment, independently and without access to the data, developed a list of select ICD-9 codes that could conceivably indicate a diagnosis of LD or ADHD, respectively. The LD list included 38 unique ICD-9 codes and ADHD list included 34 unique codes (additional file 1).

The second factor is whether the frequency of an ICD-9 code appearing in a child’s record is incorporated into the algorithm, or whether just the presence of at least one appearance of that code is considered. Repeated coding may imply that that code should be more heavily weighted, but the vagaries of the coding process may also intrude. As this would likely reduce external validity as results may be tuned to the coding practice at our institution, we decided to only pursue the approach using indication of the ICD-9 code rather than the frequency.

An ICD-9 code of 314.XX indicates a diagnosis of ADHD and has been shown in previous studies to have a high sensitivity in identification of ADHD cases when only medical records were examined [8, 12]. Therefore, a model containing a single ICD code of 314.XX was also evaluated in this study. Similar analysis was not performed for LD due to the lack of clear ICD-9 codes labeling LD.

Classification metrics of the resulting machine learning models included sensitivity, specificity, positive predictive value (PPV), negative predictive value (NPV), and overall accuracy. Kappa was estimated for overall accuracy, controlling for the expected accuracy. Concordance is also described based on the numeric predicted probability of case status. We determined a priori that to identify children with these disorders from administrative data, a model with high sensitivity and PPV would be preferred.

In the derivation cohort, results are presented using an internal non-parametric bootstrap approach. Only select models demonstrating strong sensitivity and PPV in the derivation cohort were carried forward to the validation cohort. Analyses were performed using R statistical software (R version 3.6.1) with the caret package which acts as a wrapper for functions in the glmnet, gbm, and rpart packages [13–17].

## Results

### Characteristics of study cohorts

The derivation cohort consisted of 1057 children who were born from 1996 to 2000, including 617 (58%) boys and 440 (42%) girls. The median (Q1, Q3) age at the time of code ascertainment was 16 (14–17) years. Among the study cohort of 1057 children, 175 (17%) children had a diagnosis of ADHD and 142 (13%) children had a diagnosis of LD as identified by previously established research criteria. The validation cohort consisted of 536 children who were born from 2003 to 2007, including 296 (55%) boys and 240 (45%) girls. The median (Q1, Q3) age at the time of code ascertainment was 9 (8–10) years. Among the validation cohort of 536 children, 62 (12%) children had a diagnosis of ADHD and 20 (4%) children had a diagnosis of LD.

### ADHD

In the derivation cohort, the sensitivity and PPV for models using all ICD-9 codes showed little variation according to the four classification methods examined for a given prior probability (results from validation models not presented here can be found in additional file 2-table 1). The ENET-MIN with a prior probability of 0.25 was chosen as performing well, with a sensitivity of 0.94 and a PPV of 0.84 (Table 1). In brief, sensitivity of 0.94 suggests that of those with ADHD, the model correctly classified 94%. A PPV of 84% suggests that among persons who the model classifies as having ADHD, 84% actually have ADHD. Applying only selected ICD-9 codes to this model reduced sensitivity, specificity, accuracy, and PPV (Table 1). This same pattern was observed for the other classification methods when comparing models using all vs. selected ICD-9 codes. The model using a single ICD code to identify ADHD also performed well with sensitivity and PPV greater than the selected code ENET-MIN method (Table 1).

**Table 1:** Performance of selected models in predicting attention deficit hyperactivity disorder diagnosis in the derivation and validation cohorts

	Sensitivity	Specificity	Accuracy	PPV	NPV	Kappa	Concordance
ENET-MIN all codes^a^							
Derivation cohort	0.94	0.99	0.98	0.84	0.99	0.88	0.99
Validation cohort	0.69	0.99	0.96	0.93	0.96	0.77	0.93
ENET-MIN selected codes^a^							
Derivation cohort	0.82	0.97	0.96	0.69	0.99	0.73	0.93
Validation cohort	0.76	0.98	0.96	0.85	0.97	0.78	0.91
Single code							
Derivation cohort	0.90	0.96	0.95	0.82	0.98	0.83	0.93
Validation cohort	0.81	0.98	0.96	0.83	0.97	0.80	0.89

*PPV* positive predictive value, *NPV* negative predictive value, *ENET-MIN* Elastic Net model with tuning parameters minimizing cross-validation mean misclassification error

^a^prior probability for models set at 0.25

In the validation cohort, compared with the derivation cohort sensitivity was decreased and PPV was increased for the ENET-MIN model, both for the all codes and selected codes methods (Table 1). This same pattern of results was observed for the single code method, although there was little difference in the PPV between derivation and validation cohorts.

### LD

In the derivation cohort, as in the case of ADHD the sensitivity and PPV for models using all ICD-9 codes showed little variation according to the four classification methods examined for a given prior probability (results from other validation models can be found in additional file 2-table 2). The ENET-MIN method with a prior probability of 0.4 was chosen as performing well when all codes were considered, with a sensitivity of 0.90 and a PPV of 0.72 (Table 2). Applying only selected ICD-9 codes to this model reduced sensitivity, specificity, accuracy, and PPV (Table 2), a pattern also observed for other classification methods. In the validation cohort, the ENET-MIN method performed poorly, with a sensitivity of 0.25 and a PPV of 0.12.

**Table 2:** Performance of selected models in predicting learning disability diagnosis in the derivation and validation cohorts

	Sensitivity	Specificity	Accuracy	PPV	NPV	Kappa	Concordance
ENET-MIN all codes^a^							
Derivation cohort	0.90	0.97	0.97	0.72	0.99	0.78	0.99
Validation cohort	0.25	0.93	0.90	0.12	0.97	0.12	0.72
ENET-MIN selected codes^a^							
Derivation cohort	0.59	0.96	0.93	0.55	0.97	0.53	0.81
Validation cohort	0.40	0.89	0.88	0.13	0.97	0.14	0.59

*PPV* positive predictive value, *NPV* negative predictive value, *ENET-MIN* Elastic Net model with tuning parameters minimizing cross-validation mean misclassification error

^a^prior probability for models set at 0.40

## Discussion

The main findings of the study are 1) complex machine learning models using clinical diagnosis codes perform well in identifying ADHD cases but do not offer significant advantage over a simple model using a single ICD-9 code for ADHD, and 2) clinical diagnostic codes are of limited utility in ascertaining LD cases.

Several previous studies evaluated the concordance between case ascertainment of ADHD using diagnostic codes with ascertainment using manual clinical records review, the latter using a variety of criteria to define ADHD. Gruschow et al. employed this design to review the medical records of patients with ICD-9 codes of 314.XX and a random sample of patients without this code among children who resided in New Jersey and received primary care from a single hospital network [8]. One in five of the patients who had records indicating a positive ICD code were determined to have an unknown case status upon manual review of the medical record. Depending on how ADHD diagnoses were assigned to those with unknown status, this single code method demonstrated sensitivity ranging from 0.96 to 0.97, specificity form 0.98 to 0.99, and PPV from 0.83 to 0.98. Other studies which examined only children with ADHD diagnostic codes also found that diagnostic codes reflected other information contained within the medical record with reasonable accuracy [7, 12]. Thus, if the clinical record is utilized as the basis for ADHD case ascertainment, it appears utilizing diagnostic codes can obviate the need for manual record review. However, none of these studies had access to school records, and none applied research criteria (such as excluding the diagnosis if other conditions were present as specified in DSM criteria) to define cases of ADHD. Prior work suggests that both of these factors are important to accurate ascertainment of ADHD [10, 18]. For example, approximately 11% of children in a population-based birth cohort with ADHD using the same criteria as in the current work did not have a clinical diagnosis of ADHD in their medical records [10].

Our analysis using a single diagnostic code as the criteria for ADHD ascertainment is most directly comparable to prior work. We find similarly high specificity (with correspondingly high NPV), showing that children without the ADHD diagnostic code are very unlikely to have ADHD. We also find that some children with the ADHD diagnostic code do not meet ADHD criteria, with PPV values consistent with Gruschow et al. if those with unknown status in their study are assumed to not have ADHD. The major difference from the prior work is the lower sensitivity in the current analysis, which likely reflects that some ADHD cases are identified through school procedures and are not reflected in the medical record. When taken together with prior work, these results suggest that study designs relying only on information from the medical record may underestimate the prevalence of ADHD. This limitation should be acknowledged in studies using such methods.

The availability of all diagnostic codes in our dataset and of machine learning algorithms provides the potential for improving case ascertainment by analyzing not just the occurrence of a single family of diagnostic codes [19]. As perhaps expected, models including all diagnostic codes performed the best in the derivation cohorts, but there was a greater discrepancy between derivation and validation models that included all diagnostic codes. However, we found no evidence that the use of more sophisticated models improved performance compared with using a single code. This finding suggests that there is not a practical advantage to using such models in studies using diagnostic codes to ascertain ADHD.

In contrast to ADHD, the use of diagnostic codes in machine learning methods did not prove useful in ascertaining LD. Although it was possible to produce a model with good performance using all codes for the derivation cohort, performance was poor in the validation cohort. Performance was not as good in models including selected codes, and was degraded in the validation cohort. This finding shows that as a practical matter, is it not feasible to utilize diagnostic codes available in the medical record to ascertain LD cases. LD is typically diagnosed within the educational system, and it appears that these diagnoses are not accurately reflected in the medical record.

Although complete access to all school and medical records in a geographically-defined population is a strength of this analysis, it also has several limitations. The study population includes one county in Minnesota that may not be representative of the United State population, with care provided by two healthcare systems, so that the results may not generalize to other settings. Criteria such as DSM definitions and clinical practices used to diagnose both ADHD and LD continue to evolve, have engendered some controversies [20, 21], and may differ in other settings. Diagnostic coding systems also change with time. For example, models using ICD-10 or 11 codes might have different results than the ones using ICD-9 codes. This represents a challenge to all investigation in this area [22]. The age of children in the validation cohort was substantially younger than the training cohort and those in our dataset who were listed as no LD or no ADHD could have been diagnosed with LD or ADHD subsequent to inclusion in the validation cohort. The impact to the ADHD analysis may be small as ADHD is typically diagnosed prior to the age of the validation cohort; however, LD is less clear and it could more often be diagnosed at higher ages. This may be a possible explanation for the poor validation characteristics of the LD classifier. Finally, the number of children analyzed was relatively small in comparison with other datasets used to develop machine learning algorithms, especially for the validation cohort. It is possible that model performance would improve if more children were included. However, the great amount of effort needed to review school records poses logistical barriers to analyze larger cohorts in a similar fashion.

## Conclusions

In conclusion, it is feasible to utilize medical record diagnostic codes to ascertain cases of ADHD in a population, recognizing inherent limitations in sensitivity (as not all cases are noted in the medical record) and in PPV (as not every coded diagnosis can be supported by other information in the medical record). Machine learning approaches do not have advantages compared with simply using a single family of diagnostic codes for ADHD. The use of medical record diagnostic codes is not feasible to ascertain LD cases.

## Supplementary Information

**Additional file 1.** Expert selected ICD-9 codes for ADHD.

**Additional file 2:** **Table S1.** Performance of selected models in predicting attention deficit hyperactivity disorder diagnosis in the derivation cohort. **Table S2.** Performance of selected models in predicting learning disability diagnosis in the derivation cohort

## Data Availability

The datasets generated and/or analysed during the current study are not publicly available due small geographic proximity of subjects and sensitive mental health outcomes that risks possible re-identification, but may be available from the corresponding author on reasonable request.

## Abbreviations

ADHD: Attention-deficit/hyperactivity disorder
CART: Classification trees
DSM-IV: Diagnostic and Statistical Manual of Mental Disorders-fourth edition
ENET: Elastic Net
GBM: Stochastic Gradient Boosting
ICD: International Classification of Diseases
ICU: Intensive care unit
IQ: Intelligence quotient
LASSO: Least Absolute Shrinkage and Selection Operator
LD: Learning disabilities
MIN: Minimizing cross-validation mean misclassification error
NPV: Negative predictive value
PPV: Positive predictive value
SE: Standard error
