# Supplementary material for: Utility of medical record diagnostic codes to ascertain attention-deficit/hyperactivity disorder and learning disabilities in populations of children
Source: BMC Pediatr. 2020 Nov 7;20:510. doi: 10.1186/s12887-020-02411-3 (PMC7648408; doi:10.1186/s12887-020-02411-3)
Supplement: Supplementary file 1 — Additional file 1. Expert selected ICD-9 codes for ADHD. [file 12887_2020_2411_MOESM1_ESM.docx]

Expert selected ICD-9 codes for ADHD

| Code | Description |
| --- | --- |
| 309.24 | ADJUSTMENT REACTION WITH ANXIOUS MOOD |
| 309.3 | ADJUSTMENT REACTION WITH PREDOMINANT DISTURBANCE OF CONDUCT |
| 309.4 | ADJUSTMENT REACTION WITH MIXED DISTURBANCE OF EMOTIONS AND CONDUCT |
| 309.81 | PROLONGED POSTTRAUMATIC STRESS DISORDER |
| 309.9 | UNSPECIFIED ADJUSTMENT REACTION |
| 312.00 | UNDERSOCIALIZED CONDUCT DISORDER, AGGRESSIVE TYPE, UNSPECIFIED DEGREE |
| 312.10 | UNDERSOCIALIZED CONDUCT DISORDER, UNAGGRESSIVE TYPE, UNSPECIFIED DEGREE |
| 312.23 | SOCIALIZED CONDUCT DISORDER, SEVERE DEGREE |
| 312.39 | OTHER DISORDERS OF IMPULSE CONTROL |
| 312.81 | OTHER SPECIFIED DISTURBANCES OF CONDUCT, NOT ELSEWHERE CLASSIFIED- CONDUCT DISORDER, CHILDHOOD ONSET TYPE |
| 312.89 | OTHER SPECIFIED DISTURBANCES OF CONDUCT, NOT ELSEWHERE CLASSIFIED- OTHER CONDUCT DISORDER |
| 312.9 | UNSPECIFIED DISTURBANCE OF CONDUCT |
| 313.89 | OTHER EMOTIONAL DISTURBANCES OF CHILDHOOD OR ADOLESCENCE |
| 313.9 | UNSPECIFIED EMOTIONAL DISTURBANCE OF CHILDHOOD OR ADOLESCENCE |
| 314.00 | ATTENTION DEFICIT DISORDER OF CHILDHOOD WITHOUT MENTION OF HYPERACTIVITY |
| 314.01 | ATTENTION DEFICIT DISORDER OF CHILDHOOD WITH HYPERACTIVITY |
| 314.1 | HYPERKINESIS OF CHILDHOOD WITH DEVELOPMENTAL DELAY |
| 314.9 | UNSPECIFIED HYPERKINETIC SYNDROME OF CHILDHOOD |
| V40.0 | MENTAL AND BEHAVIORAL PROBLEMS WITH LEARNING |
| V40.1 | MENTAL AND BEHAVIORAL PROBLEMS WITH COMMUNICATION (INCLUDING SPEECH) |
| V40.3 | OTHER BEHAVIORAL PROBLEMS |
| V40.9 | UNSPECIFIED MENTAL OR BEHAVIORAL PROBLEM |
| V41.0 | PROBLEMS WITH SIGHT |
| V41.2 | PROBLEMS WITH HEARING |
| V41.9 | UNSPECIFIED PROBLEM WITH SPECIAL FUNCTIONS |
| V71.02 | OBSERVATION OF CHILDHOOD OR ADOLESCENT ANTISOCIAL BEHAVIOR |
| V71.09 | OBSERVATION OF OTHER SUSPECTED MENTAL CONDITION |
| V79.3 | SCREENING FOR DEVELOPMENTAL HANDICAPS IN EARLY CHILDHOOD |
| 309.23 | SPECIFIC ACADEMIC OR WORK INHIBITION |
| 313.81 | OPPOSITIONAL DISORDER OF CHILDHOOD OR ADOLESCENCE |
| 307.9 | OTHER AND UNSPECIFIED SPECIAL SYMPTOMS OR SYNDROMES, NOT ELSEWHERE CLASSIFIED |
| 308.3 | OTHER ACUTE REACTIONS TO STRESS |
| 308.9 | UNSPECIFIED ACUTE REACTION TO STRESS |
| 309.0 | ADJUSTMENT REACTION WITH BRIEF DEPRESSIVE REACTION |

Expert selected ICD-9 codes for LD

| Code | Description |
| --- | --- |
| 294.9 | UNSPECIFIED PERSISTENT MENTAL DISORDERS DUE TO CONDITIONS CLASSIFIED ELSEWHERE |
| 315.00 | DEVELOPMENTAL READING DISORDER, UNSPECIFIED |
| 315.1 | DEVELOPMENTAL ARITHMETICAL DISORDER |
| 315.2 | OTHER SPECIFIC DEVELOPMENTAL LEARNING DIFFICULTIES |
| 315.3 | DEVELOPMENTAL SPEECH OR LANGUAGE DISORDER |
| 315.31 | DEVELOPMENTAL LANGUAGE DISORDER |
| 315.32 | RECEPTIVE LANGUAGE DISORDER (MIXED) |
| 315.39 | OTHER DEVELOPMENTAL SPEECH DISORDER |
| 315.4 | DEVELOPMENTAL COORDINATION DISORDER |
| 315.5 | MIXED DEVELOPMENT DISORDER |
| 315.8 | OTHER SPECIFIED DELAYS IN DEVELOPMENT |
| 315.9 | UNSPECIFIED DELAY IN DEVELOPMENT |
| 317 | MILD MENTAL RETARDATION |
| 319 | UNSPECIFIED MENTAL RETARDATION |
| 348.8 | OTHER CONDITIONS OF BRAIN |
| 388.40 | ABNORMAL AUDITORY PERCEPTION, UNSPECIFIED |
| 780.57 | UNSPECIFIED SLEEP APNEA |
| 783.42 | DELAYED MILESTONES |
| 784.3 | APHASIA |
| 784.49 | OTHER VOICE DISTURBANCE |
| 784.5 | OTHER SPEECH DISTURBANCE |
| 784.60 | SYMBOLIC DYSFUNCTION, UNSPECIFIED |
| 784.69 | OTHER SYMBOLIC DYSFUNCTION |
| V11.8 | PERSONAL HISTORY OF OTHER MENTAL DISORDERS |
| V57.3 | CARE INVOLVING SPEECH THERAPY |
| V79.8 | SCREENING FOR OTHER SPECIFIED MENTAL DISORDERS AND DEVELOPMENTAL HANDICAPS |
| V79.9 | SCREENING FOR UNSPECIFIED MENTAL DISORDER AND DEVELOPMENTAL HANDICAP |
| V40.0 | MENTAL AND BEHAVIORAL PROBLEMS WITH LEARNING |
| V40.1 | MENTAL AND BEHAVIORAL PROBLEMS WITH COMMUNICATION (INCLUDING SPEECH) |
| V40.3 | OTHER BEHAVIORAL PROBLEMS |
| V40.9 | UNSPECIFIED MENTAL OR BEHAVIORAL PROBLEM |
| V41.0 | PROBLEMS WITH SIGHT |
| V41.2 | PROBLEMS WITH HEARING |
| V41.9 | UNSPECIFIED PROBLEM WITH SPECIAL FUNCTIONS |
| V71.02 | OBSERVATION OF CHILDHOOD OR ADOLESCENT ANTISOCIAL BEHAVIOR |
| V71.09 | OBSERVATION OF OTHER SUSPECTED MENTAL CONDITION |
| V79.3 | SCREENING FOR DEVELOPMENTAL HANDICAPS IN EARLY CHILDHOOD |
| 309.23 | SPECIFIC ACADEMIC OR WORK INHIBITION |
| 307.9 | OTHER AND UNSPECIFIED SPECIAL SYMPTOMS OR SYNDROMES, NOT ELSEWHERE CLASSIFIED |
