# Supplementary material for: Utility of medical record diagnostic codes to ascertain attention-deficit/hyperactivity disorder and learning disabilities in populations of children
Source: BMC Pediatr. 2020 Nov 7;20:510. doi: 10.1186/s12887-020-02411-3 (PMC7648408; doi:10.1186/s12887-020-02411-3)
Supplement: Supplementary file 2 — Additional file 2: Table S1. Performance of selected models in predicting attention deficit hyperactivity disorder diagnosis in the derivation cohort. Table S2. Performance of selected models in predicting learning disability diagnosis in the derivation cohort [file 12887_2020_2411_MOESM2_ESM.docx]

**Additional file 2**

Table 1: Performance of selected models* in predicting attention deficit hyperactivity disorder diagnosis in the derivation cohort

|  | **Sensitivity** | **Specificity** | **Accuracy** | **PPV** | **NPV** | **Kappa** | **Concordance** |
| --- | --- | --- | --- | --- | --- | --- | --- |
| GBM  All codes | 0.83 | 0.97 | 0.96 | 0.70 | 0.99 | 0.74 | 0.98 |
| GBM  Selected codes | 0.81 | 0.97 | 0.96 | 0.68 | 0.98 | 0.71 | 0.92 |
| CART-1SE  All codes | 0.86 | 0.97 | 0.96 | 0.68 | 0.99 | 0.73 | 0.91 |
| CART-1SE  Selected codes | 0.86 | 0.97 | 0.96 | 0.68 | 0.99 | 0.73 | 0.91 |
| CART-MIN  All codes | 0.91 | 0.98 | 0.97 | 0.76 | 0.99 | 0.81 | 0.97 |
| CART-MIN  Selected codes | 0.86 | 0.97 | 0.96 | 0.68 | 0.99 | 0.73 | 0.91 |
| LASSO-1SE  All codes | 0.75 | 0.97 | 0.95 | 0.67 | 0.98 | 0.69 | 0.86 |
| LASSO-1SE  Selected codes | 0.75 | 0.97 | 0.95 | 0.67 | 0.98 | 0.69 | 0.86 |
| LASSO-MIN  All codes | 0.86 | 0.97 | 0.97 | 0.74 | 0.99 | 0.78 | 0.98 |
| LASSO-MIN  Selected codes | 0.82 | 0.97 | 0.96 | 0.68 | 0.99 | 0.72 | 0.92 |
| ENET-1SE  All codes | 0.75 | 0.97 | 0.95 | 0.67 | 0.98 | 0.69 | 0.95 |
| ENET-1SE  Selected codes | 0.79 | 0.97 | 0.96 | 0.68 | 0.98 | 0.70 | 0.92 |

*prior probability for models set at 0.25

GBM: Stochastic Gradient Boosting

CART: Classification trees

SE: standard error

LASSO: Least Absolute Shrinkage and Selection Operator

ENET: Elastic Net

Table 2: Performance of selected models* in predicting learning disability diagnosis in the derivation cohort

|  | **Sensitivity** | **Specificity** | **Accuracy** | **PPV** | **NPV** | **Kappa** | **Concordance** |
| --- | --- | --- | --- | --- | --- | --- | --- |
| GBM  All codes | 0.58 | 0.97 | 0.94 | 0.59 | 0.97 | 0.55 | 0.93 |
| GBM  Selected codes | 0.50 | 0.96 | 0.92 | 0.50 | 0.96 | 0.46 | 0.78 |
| CART-1SE  All codes | 0.62 | 0.93 | 0.90 | 0.42 | 0.97 | 0.45 | 0.78 |
| CART-1SE  Selected codes | 0.52 | 0.92 | 0.89 | 0.36 | 0.96 | 0.37 | 0.73 |
| CART-MIN  All codes | 0.94 | 0.89 | 0.90 | 0.43 | 0.99 | 0.53 | 0.93 |
| CART-MIN  Selected codes | 0.53 | 0.96 | 0.92 | 0.51 | 0.96 | 0.48 | 0.79 |
| LASSO-1SE  All codes | 0.62 | 0.93 | 0.90 | 0.42 | 0.97 | 0.45 | 0.83 |
| LASSO-1SE  Selected codes | 0.57 | 0.95 | 0.92 | 0.51 | 0.96 | 0.50 | 0.80 |
| LASSO-MIN  All codes | 0.89 | 0.97 | 0.96 | 0.70 | 0.99 | 0.76 | 0.98 |
| LASSO-MIN  Selected codes | 0.57 | 0.95 | 0.92 | 0.51 | 0.96 | 0.50 | 0.80 |
| ENET-1SE  All codes | 0.60 | 0.98 | 0.95 | 0.71 | 0.97 | 0.62 | 0.92 |
| ENET-1SE  Selected codes | 0.59 | 0.96 | 0.93 | 0.54 | 0.96 | 0.52 | 0.80 |

*prior probability for models set at 0.40
